# Proteomic analysis of stromal proteins in different stages of colorectal cancer establishes Tenascin-C as a stromal biomarker for colorectal cancer metastasis

**DOI:** 10.18632/oncotarget.9362

**Published:** 2016-05-14

**Authors:** Maoyu Li, Fang Peng, Guoqing Li, Yang Fu, Ying Huang, Zhuchu Chen, Yongheng Chen

**Affiliations:** ^1^ Key Laboratory of Cancer Proteomics of Chinese Ministry of Health, Xiangya Hospital, Central South University, Changsha, 410008, Hunan Province, China; ^2^ Molecular and Computational Biology Program, Department of Biological Sciences, University of Southern California, Los Angeles, CA 90089, USA; ^3^ Maternal and Child Health Hospital of Hunan Province, Changsha, 410008, Hunan Province, China; ^4^ Collaborative Innovation Center for Cancer Medicine, Guangzhou, 510060, Guangdong, China

**Keywords:** colorectal carcinoma, multistage carcinogenesis, stromal biomarker, quantitative proteomics, Tenascin-C

## Abstract

Tumor microenvironment is crucial to tumor development and metastasis. Little is known about the roles of stromal proteins in colorectal carcinogenesis. In this study, we used a combination of laser capture microdissection (LCM), iTRAQ labeling and two-dimensional liquid chromatography-tandem mass spectrometry (2D LC-MS/MS) to compare stromal proteomes in different stages of colorectal cancer. A total of 1966 proteins were identified, and 222 proteins presenting a significant fold change were quantified in different stages. Differentially expressed proteins (DEPs) were subjected to cluster and pathway analyses. We confirmed the differential expression of Tenascin-C and S100A9 using immunohistochemical analysis, and found that the expression levels of S100A9 and Tenascin-C were correlated with TNM stages and metastasis. In addition, our results showed that Tenascin-C was abundantly secreted by the colon cancer cells with high metastatic potential, and highly expressed in lymph nodes with metastasis. Our studies not only shed light on the mechanism by which stromal proteins contributed to colorectal carcinogenesis, but also identified Tenascin-C as a potential stromal biomarker for colorectal cancer metastasis.

## INTRODUCTION

Colorectal cancer (CC) is the third most common type of cancer, affecting over a million people worldwide per year [1]. CC is also one of the most lethal malignancies, and 5-years survival rate for patients with metastasis is extremely low [2]. Early diagnosis of CC can improve survival rate, however, most patients are diagnosed at an advanced stage. Therefore, identification of biomarkers for early diagnosis is essential for improving survival of the CC patients [3].

The genetic mechanisms of colorectal carcinogenesis are extensively studied [4, 5]. Previous studies have been focused on the cancer cells and oncogenes/tumor suppressors, such as p53 [6, 7], c-myc [8], and epithelial growth factor receptor (EGFR) [9, 10]. These proteins were considered as targets for drug development [11]. However, only limited success was achieved by only targeting cancer cells. More and more evidences suggested that cancer is a disease involving a dynamic interplay between cancer cells and surrounding stromal cells [12]. Normal microenvironment plays important roles to maintain tissue architecture, inhibit cell growth, and constrain the tumor growth [13]. Cancer cells exposed to a normal microenvironment could lose their malignant behaviors [14, 15]. However, cancer cells can alter their adjacent stroma to form a permissive and supportive environment for tumor progression [16].

Colorectal carcinogenesis is a multistage process, which originated from normal mucosa (NM), and then adenomatous polyps (adenoma) (AP), carcinoma in situ (CIS), and ultimately to invasive and metastatic carcinoma (IC) [5]. Recent study compared the protein expressions between normal and malignant colonic stroma [17]. However, there has been no systematic comparison of stromal proteomes among multiple stages of colorectal cancer, and little is known about the dynamic alterations at the proteome level during the colorectal carcinogenesis.

In the present study, a combination of laser capture microdissection (LCM), iTRAQ labeling, and two-dimensional liquid chromatography-tandem mass spectrometry (2D LC-MS/MS) was used to study stromal proteomes in different stages of colorectal cancer. LCM was used to collect the stroma from four stages of colorectal cancer tissues, and iTRAQ based quantitative proteomics was used to identify differentially expressed proteins (DEPs) in different stages. A total of 222 DEPs were found among different stages. The expression dynamics of these DEPs was further analyzed and subjected to cluster and pathway analyses. Two of the top-ranked DEPs (Tenascin-C and S100A9) were further validated by immunohistochemistry. Our studies also showed that the expression levels of S100A9 and Tenascin-C were correlated with TNM stages and metastasis. In addition, we found that Tenascin-C was abundantly secreted by the colon cancer cells with high metastatic potential, and highly expressed in lymph nodes with metastasis.

## RESULTS

### Identification and quantitation of stromal proteome in different stages of colorectal cancer

To study the stromal proteomes in different stages of colorectal cancer, the stroma from four clinical stages of carcinogenesis were purified using laser capture microdissection (LCM) (Supplementary Figure S1), and subjected to three 8-plex iTRAQ experiments. A total of 1966 non-redundant proteins were identified at a minimum confidence level of 95% (unused ProtScore> 1.3) in three iTRAQ experiments, among which 1138 (57.9%) proteins were identified in each of the three experiments, whereas 303 (15.2%) were common to at least two experiments. This result indicated that nearly three-fourths of the identified proteins could be detected in at least two of the three experiments (Supplementary Figure S2A).

Out of the identified proteins, 1881 (94.2%) proteins were quantified, in which 1395 (74.2%) proteins were quantified in more than two iTRAQ experiments (Supplementary Figure S2B). Using the concatenated target-decoy database search strategy as detailed by Elias and Gygi [18], a 1.03%, 1.02% and 0.55% rate of false positives was estimated for the three iTRAQ experiments respectively.

All identified proteins were classified according to GO term at the biological process, molecular function and cellular compartment level, using PANTHER GO classification system. Based on the subcellular distribution, the identified proteins included cytoplasmic proteins (35.4%), organelle proteins (26.6%), macromolecular complex proteins (17.4%), extracellular region proteins (8.3%), and membrane proteins (5.3%) (Supplementary Figure S2E). The identified proteins were associated with a broad range of biological processes and molecular functions (Supplementary Figure S2).

### Determination of cutoff threshold for significant fold-changes in iTRAQ experiments

**Figure 1:**
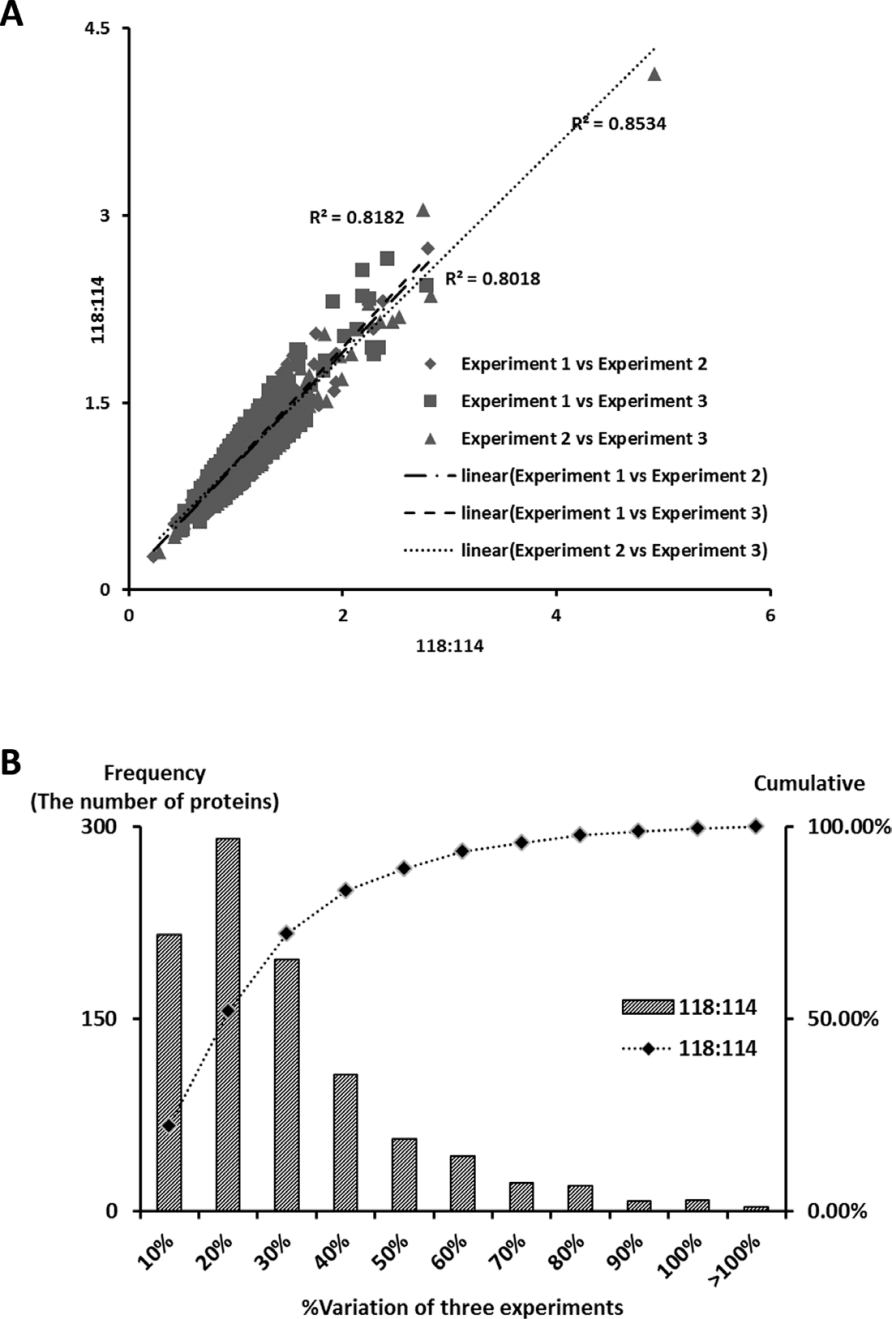
Correlation between iTRAQ experiments and determination of cutoff value for significant fold-change **A.** Plotting of iTRAQ ratios (118/114) between the three iTRAQ experiments. The 405, 373, 401 commonly quantified proteins in the iTRAQ experiments (#1 versus #2, #1 versus #3 and #2 versus #3) were plotted in the linear dynamic range. The experimental variations yielded a correlation coefficient of r2 = 0.8018 (experiment #1 vs. #2), r2 = 0.8181 (#1 vs. #3), and r2 = 0.8534 (#2 vs #3), respectively. **B.** The % variations among the 3 iTRAQ ratios for the common proteins in the 3 iTRAQ experiments. The commonly quantified proteins in the 3 iTRAQ experiments were input to calculate % variations. The vertical axis represents the number of proteins, and the horizontal axis denotes % variation. The % variation was rounded off to the nearest number. The right vertical axis represents the cumulative % of the counted proteins. Ninety percent of the counted proteins fell within a variation of 50%. Therefore, a fold-change ≥ 1.5 or ≤ 0.66 is a sufficient cutoff that reflects significant changes in the 3 iTRAQ experiments.

According to previous studies, the variations in iTRAQ experiments are composed of technical, experimental and biological variations. In the present study, the biological variation was minimized by sample pooling effect. The remained variations predominantly were resulted by experimental errors, which could be determined by using the experimental replicates method. Accordingly, 405 proteins commonly quantified in the three iTRAQ experiments were selected based on the following selection criteria: unused score > 1.3, more than 2 unique peptides (>95%) contained, p-value<0.05 and EF<2. Experimental variations for the 118/114 reporter ions were calculated using the ratios of the 405 common quantified proteins among the three iTRAQ experiments. The experimental variations were r^2^=0.8182 (Experiment 1 vs Experiment 2), r^2^=0.8018 (Experiment 1 vs Experiment 3), and r^2^=0.8534 (Experiment 2 vs Experiment 3), which indicate that the results are reproducible (Figure 1A). Therefore, these proteins were used to determine the experimental variations and to confirm the cutoff for meaningful fold-changes. Consequently, 90% of the identified proteins in the 3 iTRAQ experiments fell within 50% of the respective experimental variation (Figure 1B) and fold-changes > 1.5 or < 0.67 were determined as a threshold.

### Differentially expressed proteins in different stages of colorectal cancer

To identify the DEPs in the colon carcinogenic process, protein expression profiles of ACP, CCIS or ICC and NNCM were compared. The proteins were considered as DEPs according to the criteria as described in Materials and Methods section, and the fold change cutoff was >1.5 or <0.67. A total of 222 DEPs were found, among which 199 proteins were found in ACPS vs NNCMS, 185 proteins in CCIS vs NNCMS, and 174 proteins in ICCS vs NNCMS. Top 10 differentially expressed proteins (up-regulated and down-regulated) between the ICCS and NNCMS were listed in Table 1. Out of 222 DEPs, 131 (59%) proteins expression levels were significantly altered in the three pathological stages (Supplementary Figure S3A). Based on Uniprot annotations, most of proteins belong to membrane or extracellular matrix proteins (Supplementary Figure S3B).

**Table 1:** Top 10 differentially expressed proteins (up-regulated and down-regulated) between the ICCS and NNCMS

No	Gene Symbol	Protein Name	ACPS/NNCMS	CCISS/NNCMS	ICCS/NNCMS
1	S100A9	Protein S100-A9	10.26	7.13	18.71
2	MYH9	Myosin-9	3.94	11.12	13.51
3	TNC	Tenascin-C	2.14	23.99	12.71
4	HSPA5	78 kDa glucose-regulated protein	8.42	6.56	10.81
5	FGB	Fibrinogen beta chain	0.21	0.73	10.57
6	FGG	Fibrinogen gamma chain	0.21	0.59	9.32
7	EPX	Eosinophil peroxidase	16.67	16.07	8.78
8	S100A8	Protein S100-A8	9.35	6.14	8.63
9	RRBP1	Ribosome-binding protein 1	4.97	5.8	8.07
10	FGA	Fibrinogen alpha chain	0.2	0.44	7.79
11	TPM2	Tropomyosin beta chain	0.04	0.43	0.13
12	TPM1	Tropomyosin alpha-1 chain	0.04	0.43	0.12
13	TPSAB1	Tryptase alpha/beta-1	0.16	0.09	0.12
14	CALM1	Calmodulin	1.13	0.55	0.12
15	FHL1	Four and a half LIM domains protein 1	0.09	0.12	0.12
16	PRPH	Peripherin	0.04	0.09	0.09
17	HIST1H1D	Histone H1.3	1.51	0.52	0.08
18	HIST1H1C	Histone H1.2	1.93	0.62	0.06
19	CKB	Creatine kinase B-type	0.42	0.16	0.05
20	HIST1H1E	Histone H1.4	1.15	0.50	0.02

In order to understand the characteristics of DEPs, the DEPs were annotated by GO term at the biological process, molecular function and subcellular compartment. Hypergeometric test was used to identify the GO term in which the DEPs were over-represented compared with the total identified proteins. As shown in Supplementary Figure S4A, DEPs were significantly over-represented in “developmental process” and “cellular process”. Especially, more than one third of the identified DEPs were involved in “biological adhesion”. In addition, DEPs were found to be enriched in “structural molecule activity”, “binding”, “catalytic activity”, and other molecular functions. Nearly 40% of the DEPs were involved in “receptor activity” (Supplementary Figure S4B). Interestingly, another 40% of the DEPs were found to be located in “extracellular region”, “cell junction”, and “extracellular matrix”. These results suggested that extracellular matrix or cell junction proteins might play crucial roles in colon carcinogenesis (Supplementary Figure S4C).

### Cluster analysis of differentially expressed proteomes and functional analysis

**Figure 2:**
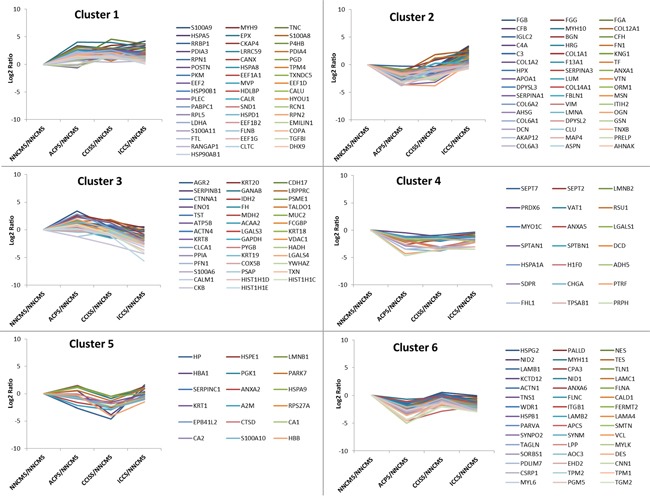
K-mean clusters of differentially expressed proteins These proteins could be clustered into six clusters. According the average tendency, the 6 clusters can be arbitrarily categorized into three groups. Group 1 includes cluster 1, in which the abundance of proteins progressively increase during the colorectal carcinogenic process. Group 2 consists of cluster 4, in which the abundance of proteins progressively reduced during the process. Group 3 consists of cluster 2, 3, 5, and 6, in which the abundance of proteins fluctuated during the process.

To know more about the expression dynamics of the DEPs during the colon carcinogenesis, k-means clustering analysis was used to group the DEPs into clusters. The 222 proteins were classified into 6 clusters (Figure 2, Supplementary Table S2). According to the overall tendency of protein expression in each cluster, the six clusters were arbitrarily categorized into three groups. Group 1 only included cluster 1, in which the abundance of proteins were increased at all the three stages: ACP, CCIS, and ICC. Group 2 consisted of cluster 4, in which the abundance of DEPs was decreased at all the three stages: ACP, CCIS, and IC. Group 3 consists of cluster 2, 3, 5, and 6, in which the abundance of proteins fluctuated during the colonic carcinogenic process.

Theoretically, the proteins in each cluster are co-regulated and may have similar biological functions during the colon carcinogenesis. Co-regulated families of genes have been shown to be clustered together in colon carcinoma tissue [19]. Cluster analysis of genome-wide expression data revealed a strong tendency for larger groups of clustered genes to share common roles in cellular processes [20]. To get more insights on the characteristics of the DEPs, they were annotated as PANTHER protein class. As shown in Supplementary Figure S5, some protein classes such as “cytoskeleton protein”, “receptor”, and “signaling molecule” existed in all the clusters, whereas other protein classes like “extracellular matrix protein”, “cell adhesion molecule” only existed in some of the clusters. In order to further understand their biological significance, signaling pathway analysis was used to identify the pathway in which the DEPs in each cluster were involved. Pathway analysis revealed that proteins involved in “endogenous TLR signaling” and multiple integrin-related signaling pathways were enriched in cluster 1. The proteins in cluster 4 were enriched in proteins involved in “regulation ras family activation” and “N-cadherin signaling events” (Supplementary Figure S6).

Since extracellular matrix proteins and secreted proteins are important components of microenvironment, we further characterized the proteins that are secreted proteins or extracellular matrix proteins. According to the Uniprot database, 35 of the DEPs were extracellular matrix proteins, and 20 were secreted proteins. (Supplementary Table S2).

### Validation of differentially expression of S100A9 and Tenascin-C using immunohistochemistry

**Figure 3:**
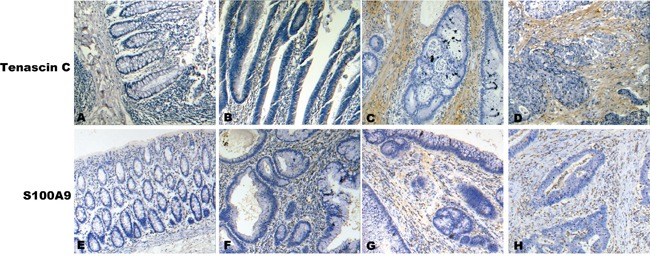
A representative result of immunohistochemistry shows the expression of TNC, S100A9 in stroma at NCM, ACP, CIS and ICC Original magnification, ×200. Top panel, TNC immunostaining of NCM **A.**, AP **B.**, CIS **C.** and IC **D.** Bottom panel, S100A9 immunostaining of NCM **E.**, ACP **F.**, CIS **G.** and ICC **H.**

Interestingly, for the two top-ranked DEPs, S100A9 is a secreted protein, and Tenascin-C (TNC) is a ECM protein. These two proteins were chosen for further validation using immunohistochemistry. Tissue specimens, including 50 cases of NNCM, 50 cases of ACP, 30 cases of CCIS, and 63 cases of ICC, were used for detecting the expressions of the two proteins by immunohistochemistry (IHC). There was a significant difference when we compared the expression levels of S100A9 and TNC in stroma of ACP, CCIS, and ICC with that in NNCM. (Mann-Whitney U test, P < 0.05; Table 2). TNC was negatively stained in the stroma of NNCM, weakly stained in the stroma of ACP, and strongly stained in the stroma of CCIS and ICC (Figure 3, top panel). S100A9 staining was much stronger in the stroma of ACP, CCIS, and ICC than that of NNCM (Figure 3, bottom panel). These results were consistent with our findings in the iTRAQ-based experiments.

**Table 2:** The expressions of two proteins(TNC, S100A9) in stroma at various stages of colonic epithelial carcinogenesis by IHC

	N	Score			p-value
		Low(0-2)	Medium(3-4)	High(5-6)	
TNC					
NNCMS	50	37	11	2	
ACPS	50	26	20	4	0.024^a^
CCISS	30	8	17	5	0.000^a^,0.024^b^
ICCS	63	10	25	28	0.000^a^,0.014^c^
S100A9					
NNCMS	50	34	13	3	
ACPS	50	15	30	5	0.000^a^
CCISS	30	4	18	8	0.000^a^,0.023^b^
ICCS	63	7	23	33	0.000^a^,0.041^c^

a compared with NNCMS

b compared with ACPS

c compared with CCISS. (p-value determined by Mann-Whitney U test)

Furthermore, we examined the correlation of the expression of these two proteins with certain clinicopathological features in the 93 cases of colon carcinoma tissues above (30 cases of CCIS and 63 cases of ICC). The results showed that the expression levels of S100A9 and TNC in colon carcinoma tissues were not correlated with age or gender (P > 0.05), but correlated with TNM stages and metastasis (P < 0.05; Table 3).

**Table 3:** Relationships between S100A9 or TNC expression and clinicopathological factors in colon carcinoma

	N	S100A9				TNC			
		Low (0-2)	Medium (3-4)	High (5-6)	P-value	Low (0-2)	Medium (3-4)	High (5-6)	P-value
Gender					0.611				0.531
Male	51	6	24	21		11	23	17	
Female	42	5	17	20		7	19	16	
Age					0.379				0.803
≥62	44	7	19	18		8	20	16	
<62	49	4	22	23		10	22	17	
TNM Stages					0.012				0.002
CIS	30	4	18	8		8	17	5	
I	9	3	4	2		3	5	1	
II	11	2	5	4		3	6	2	
III	24	1	8	15		2	9	13	
IV	19	1	6	12		2	5	12	
Metastasis					0.004				0.000
Yes	43	3	14	26		4	14	25	
No	50	8	27	15		14	28	8	

p-value determined by Mann-Whitney U test

### TNC as a potential stromal marker for colonic metastasis

**Figure 4:**
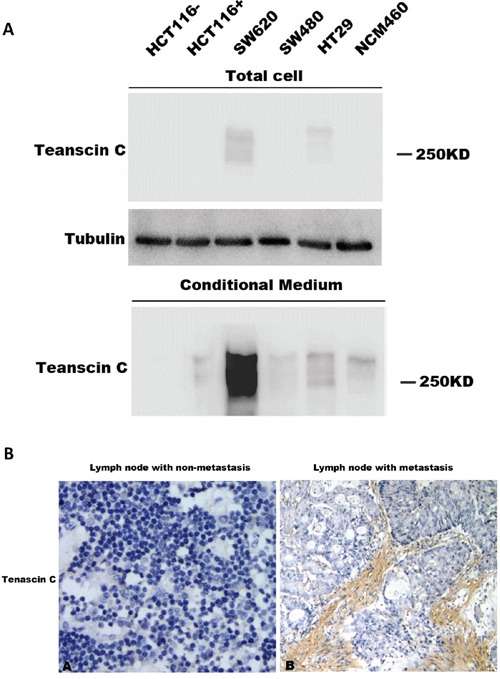
Expression of TNC in conditioned medium and its association with metastasis **A.** TNC protein level in conditioned medium from human colon carcinoma cell lines was detected by western blotting analysis. **B.** TNC protein expression in lymph node with or without metastasis was detected by IHC analysis.

Correlation analysis of the expression level of TNC with clinical characteristics demonstrated that its expression was closely correlated with metastasis (Mann-Whitney U test, p<0.05, Table 4). We further analyzed its expression level in lymph nodes. No TNC expression was detected in lymph nodes without metastasis. In 31 cases of lymph node tissues with metastasis, high expression level of TNC was observed in 17 cases (54.8%), medium expression in 9 cases, and low/no expression in 5 cases. Representative images of TNC staining in lymph nodes with or without metastasis were shown in Figure 4B.

**Table 4:** Relationships between TNC expression and lymph nodes metastasis in colon carcinoma

	N	TNC			
		Low(0-2)	Medium(3-4)	High(5-6)	P-value
Lymph Nodes					0.000
non-metastasis	10	8	2	0	
Metastasis	31	5	9	17	

p-value determined by Mann-Whitney U test

In order to test whether the carcinoma cells contribute the expression of TNC in stroma, we collected the conditioned media of colorectal carcinoma cell cultures, including HCT116, SW620, SW480, HT29, and NCM460, for western blotting analysis (Figure 4A). TNC contain a variable number of FN III domains, which result in the protein products with a molecular weight of 180-300 kDa [21]. TNC level is much higher in the conditioned medium than in the cell lysate (Figure 4A), supporting the fact that TNC is a ECM protein. Among the cell lines, SW620 cell line, a cell line with high metastatic potential, had the highest TNC level, especially in the conditioned medium (Figure 4A). Our results suggested that TNC might play an important role in colorectal carcinoma metastasis.

## DISCUSSION

Carcinogenesis is a multistage process, involving interplay between the cancer cells and their surrounding stromal cells [22–24]. Extracellular matrix proteins and secreted proteins are important components of the stroma, which could play important roles in the cell-cell communication, biological adhesion, and regulation of cell process [25]. Cancer cells may alter their stroma by secreting proteins such as cytokine, chemokine and other factors [26]. On the other hand, stromal cells can affect the phenotype, invasiveness, metastatic capacity of cancer cells [14]. However, little is known about the roles of stromal proteins in the process of colorectal carcinogenesis. Therefore, we carried out a systematic analysis of stroma proteomes in various stages of colorectal carcinogenesis. In this study, most of the DEPs belong to extracellular compartment, including extracellular matrix proteins, secreted proteins, and other extracellular components. Of the two top-ranked DEPs, S100A9 is a secreted protein, and TNC is an ECM protein.

S100A9 is a member of S100 protein family, which is involved in many cellular processes such as cell cycle progression and differentiation [27]. Under physiological conditions, the expression of S100A9 is dominantly restricted to myeloid cells, including neutrophils and monocytes [28]. S100A9 could enhance proinflammatory reaction by promoting leukocyte migration and inducing the release of cytokines and chemokines [29]. Up-regulation of S100A9 expression was observed in many tumors, including breast, colon, skin, and gastric cancers [30–33]. S100A9 is located in the cytoplasm, plasma membrane, nuclei, and can be released into the extracellular space through a Golgi-independent pathway [34]. Extracellular S100A9 could bind to various receptors such as TLR4 to activate signaling cascades and trigger cellular responses [35]. In our study, we showed that the expression level of S100A9 gradually increased from NNCM to ICC. We also showed that the expression level of S100A9 was related to TNM stages and metastasis.

TNC, an extracellular protein, contains four domains: an assembly domain, EGF-like repeats, fibronectin type III-like repeats (FNIII), and a C-terminal fibrinogen-like globe (FBG) [36]. Each of these domains can bind various partners, including cell surface receptors and other extracellular components [37]. Little or no expression of TNC was detected in normal adult tissues, but it is overexpressed in embryonic tissues, or injured tissues caused by inflammation, infection, or tumorigenesis [21]. Tumor-derived upregulation of TNC was reported to be associated with the aggressiveness of pulmonary metastasis for breast cancer [38]. High level of TNC was identified in the exosomes derived of metastatic colonic cancer cells [39]. In the present study, we found that the expression of TNC was progressively increased during colorectal carcinogenesis. We also showed that TNC was released in the conditioned medium from different colorectal carcinoma cell lines. The highest expression of TNC was observed in conditioned media of SW620 cell line, a cell line with high metastatic potential. In addition, we demonstrated that TNC upregulation was associated with lymph node metastasis. Taken together, our studies strongly suggested that TNC could be a stromal biomarker for colorectal carcinoma metastasis.

The present study investigated for the first time the dynamic expression patterns of stromal proteins in multiple stages of colorectal carcinogenesis. Our results greatly helped our understanding of the role of stromal proteins in the process of colorectal carcinogesis. In addition, our study identified an extracellular protein, Tenascin-C, as a potential stromal biomarker for colorectal carcinoma metastasis.

## MATERIALS AND METHODS

### Sample collection

Twenty-seven cases of fresh colonic tissues, including 5 cases of non-neoplastic colonic mucosa (NNCM), 8 cases of adenomatous colon polyps (ACP), 5 cases of colon carcinoma in situ (CCIS) and 9 cases of invasive colonic carcinoma (ICC), were obtained between January 2011 to December 2012 from the Department of Surgery, Xiangya Hospital, Central South University, China. The patients received neither chemotherapy nor radiotherapy before curative surgery and signed an informed consent form for the study approved by the local ethical committee. All tissue specimens were obtained from surgical resection and NNCM tissue was obtained from biopsy of patients with non-neoplastic colonic disease. All of the tissues were stored at −80°C until further use. The parameters of patients and tissue specimens are shown in Supplementary Table S1.

### Tissue processing and LCM

Four types of tissues (NNCM, ACP, CCIS and ICC) were diagnosed by pathological examination of hematoxylin and eosin stained tissue section. LCM was used to purify the stroma from NNCM, ACP, CCIS and ICC tissue, respectively, according to our previous procedure [40, 41]. Homogeneous (>95%) was determined by microscopic visualization of the captured cells (Supplementary Figure S1).

### Cell culture and conditional media preparation

The human colon carcinoma cell lines HCT116, SW620, SW480, HT29 and human normal colon cell line NCM460 were chosen for conditional media preparation. Conditioned media was collected as previously described [42] but with minor modifications. Briefly, approximately 3 × 10^6^ cells were grown to 80% confluence, washed 6 times with PBS, and incubated for 24h in serum-free DMEM. The conditional medium was collected and filtered using a 0.45-μm filter (Millipore) and subsequently concentrated using a using a Millipore centrifugal filter (3 kDa). The protein concentration was determined using a standard Bradford protein assay (Thermo Scientific). After culture medium was removed, the cell monolayer was washed twice with 10 ml of ice-cold PBS and scraped in the presence of 1 ml PBS with protease inhibitors. After removing PBS by centrifugation at 6,000 × g for 5 min at 4°C, the pellet was lysed in NP-40 buffer (20 mM Tris pH 7.5, 150 mM NaCl, 1 mM EDTA, 1% NP-40, 1:100 protease inhibitor cocktail). Protein concentration was determined using the Pierce BCA Protein Assay Kit (Thermo Scientific)

### Protein extraction and labeling with iTRAQ reagents

The microdissected samples were dissolved in lysis buffer (7 M urea, 2 M thiourea, 65mM dithiothreitol, 0.1mM phenylmethylsulfonyl fluoride) at 4°C for 1 h. After being centrifuged at 12,000 rpm for 30 min at 4°C, the supernatant was collected and the protein concentration was determined by the 2D Quantification Kit (Amersham Biosciences). For each type of tissue, equal amount of proteins from all the individual sample were mixed to generate a pooled sample. Totally, 4 pooled protein samples (corresponding to NNCM, ACP, CCIS and ICC, respectively) were obtained for iTRAQ labeling. Trypsin digestion and iTRAQ labeling were performed according to the manufacturer's protocol (Applied Biosystems). In brief, 100 μg protein of each pooled sample was reduced and alkylated, and then digested overnight at 37°C with trypsin (mass spectrometry grade; Promega). The sample was then labeled with iTRAQ™ reagents as follows: ACP Stroma (ACPS, labeled with iTRAQ 113 and 117); NNCM stroma (NNCMS, labeled with iTRAQ 114 and 118); CCIS stroma (CCISS, labeled with iTRAQ 115 and 119); and ICC stroma (ICCS, labeled with iTRAQ 116 and 121). The labeled samples were then mixed and dried. The experiments repeated thrice.

### Off-line 2D LC-MS/MS

The mixed peptides were first separated on a strong cation exchange (SCX) column into ten fractions according to the procedure described in our previous study [17]. Each fraction was dried down by the rotary vacuum concentrator, then dissolved in solvent A (5% acetonitrile, 0.1% formic acid) and analyzed on TripleTOF 5600 systems (AB SCIEX) in an information dependent mode. Briefly, peptides were separated on reverse-phase column (ZORBAX 300SB-C18 column, 5μm, 300 Å, 0.1 × 15 mm; Micromass) using an Eksigent 1D PLUS system (AB SCIEX) at an analytical flow rate of 300 nL/min. The peptides were separated with an 120 min linear gradient from 5% to 40% solvent B (0.1% formic acid/90% acetonitrile). Survey scans were acquired from 400 to 1500 with up to 15 precursors selected for MS/MS and dynamic exclusion for 20 sec.

### Data analysis

Protein identification, grouping and quantitation were performed using Paragon and Pro Group algorithm in ProteinPilot™ 4.2(Applied Biosystems). The data analysis parameters were set as follows: (1) Sample Type: iTRAQ 8plex (Peptide Labeled); (2) Cysteine Alkylation: MMTS; (3) Digestion: Trypsin; (4) Instrument: TripleTOF 5600; (5) Special Factors: None; (6) Species: Homo sapiens; (7) ID Focus: Biological modifications; (8) Database: Uniprot human database (release Apr 2013); (9) Search Effort: Thorough; (10) Max missed cleavages; (11) FDR Analysis: Yes; (12) User Modified Parameter Files: No; (13) Bias Correction: Auto; (14) Background Correction: Yes. Identified proteins were grouped by the software to minimize redundancy. All peptides used for the calculation of protein ratios were unique to the given protein or proteins within the group, and peptides that were common to other isoforms or proteins of the same family were ignored.

A decoy database search strategy was adopted to estimate the false discovery rate (FDR) for peptide identification. For our iTRAQ experiments, a strict unused confidence score cutoff is 1.3, which corresponds to a peptide confidence level of 95%. With this filter, the corresponding false discovery rate was calculated by searching against a concatenated reversed database. The results were then exported into Microsoft Excel for manual data interpretation.

For each of the three iTRAQ replicate experiments, an 8-plex iTRAQ experiments were used to compare the four pooled samples, where each pooled sample were labeled duplex(technical replicates). Thus, each protein in a pooled sample can be measured twice in an iTRAQ experiment, and up to six times in all the three experimental replicates.

In order to identify the DEPs, we adopted a stringent criteria: Briefly, 1) the proteins that are quantified in at least four out of the six times replicates; 2) change compared with normal control had to be statistically significant (p<0.05); 3) fold change had to be greater than a threshold, which was determined using the experimental replicate method as described in Results section. The average iTRAQ ratios from the replicates were calculated for each protein.

### Cluster analysis of DEPs and bioinformatics analysis

To identify co-regulated proteins, the clustering of DEPs was performed based on the log2 of the iTRAQ ratios. Total significant proteins were clustered by the k-means clustering method with MultiExperiment Viewer software (version 4) [43]. Euclidean distance was used for metrics, and k-values were seeded randomly. Silhouette plot estimation was used for the determination of the number of correct clusters (Supplementary Figure S6). The silhouette value was calculated by Orange data mining software (http://orange.biolab.si/). The proteins were annotated using PANTHER database (http://www.pantherdb.org/) [44]. A binomial test was used to find GO terms in DEPs that were significantly enriched compared with all human genes in PANTHER database [45]. The GO terms was considered statistically significant enriched when the corrected p-value less than 0.05. Pathway analysis were performed using NCI-Nature Pathway Interaction Database query tool (http://pid.nci.nih.gov), which uses a hypergeometric distribution to compute the probability that each pathway in the database is hit by proteins in the query list. The pathway with the p-value less than 0.05 was considered statistically significant enriched.

### Immunohistochemistry

An additional set of formalin-fixed and paraffin-embedded archival tissue specimens, composed of 50 cases of NNCM, 50 cases of ACP, 30 cases of CCIS, 63 cases of ICC, was obtained from the Department of Pathology of Xiangya Hospital at Central South University, used for immunohistochemical analysis according to the procedure described in our previous study [46]. Briefly, the sections were incubated with anti-TNC (1:1000; sigma) or anti-S100A9 (1:800; sigma) overnight at 4°C, and then were incubated with biotinylated secondary antibody followed by addition of avidin-biotin peroxidase. Diaminobenzidine was used as the chromogen. Finally, the sections were counterstained with hematoxylin. As a negative control, the primary antibody was omitted. The evaluation of immunostaining was performed as previously described by us [46]. A score (ranging from 0–6) was obtained for each case. A combined staining score of≤2 was considered to be negative staining (no expression); a score between 3 and 4 was considered to be moderate staining (low expression); and a score between 5 and 6 was considered to be strong staining (high expression).

### Western blot analysis

For western blot analysis, conditioned media samples or whole cell lysate samples were subjected to SDS-PAGE and subsequently transferred to PVDF membranes (Millipore). Membranes were blocked in 5% milk for two hours prior to primary antibody Rabbit anti-TNC (Santa Cruz, 1:1000) incubation overnight. Tubulin was used as a control for protein loading and was detected using a mouse anti-α-tubulin antibody (1:5000, Sigma). Membranes were incubated with the peroxidase-coupled secondary antibodies for 1 hour. After extensive washing, the membrane was visualized using ECL agent (GE Healthcare Biosciences).

### Statistical analysis

SPSS software (IBM, v19) was used for statistical analysis. A hypergeometric distribution test was used to define the PANTHER protein classes that were enriched in each cluster (hypergeometric test: P < 0.05). Non-normal distribution data were compared with Wilcoxon rank sum test (p<0.05).

## SUPPLEMENTARY FIGURES AND TABLES




