# Supplementary material for: Proteomic analysis of stromal proteins in different stages of colorectal cancer establishes Tenascin-C as a stromal biomarker for colorectal cancer metastasis
Source: Oncotarget. 2016 May 14;7(24):37226–37. doi: 10.18632/oncotarget.9362 (PMC5095071; doi:10.18632/oncotarget.9362)
Supplement: Supplementary file 1 [file oncotarget-07-37226-s001.pdf]

Proteomic analysis of stromal proteins in different stages of colorectal cancer establishes Tenascin-C as a stromal biomarker for colorectal cancer metastasis

SUPPLEMENTARY FIGURES AND TABLES

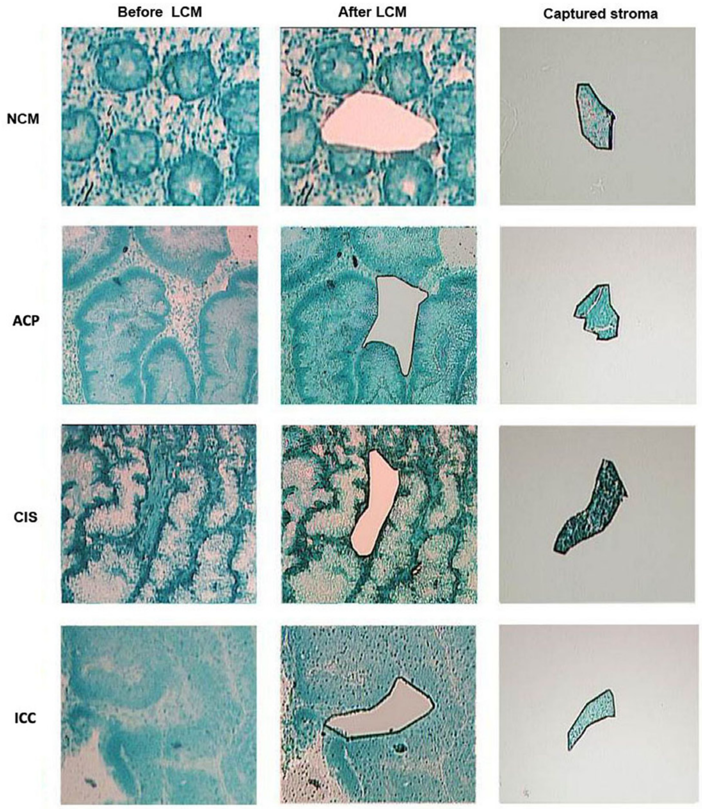

Supplementary Figure S1: Purification of stroma from different stages of human colorectal cancer tissues by LCM.

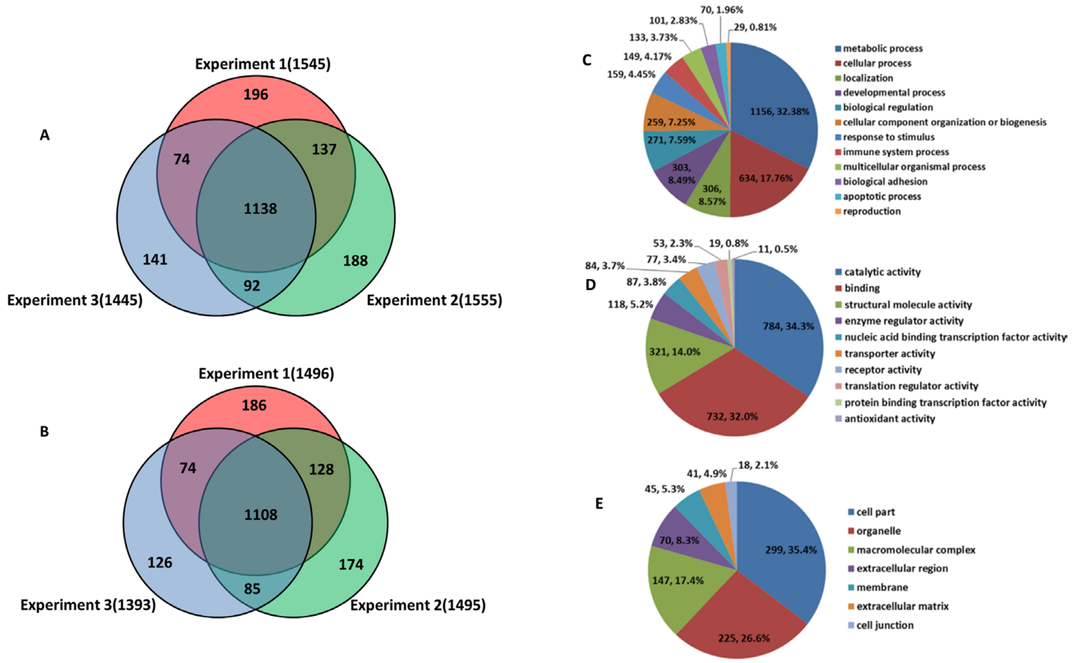

Supplementary Figure S2: Venn diagram depicting the overlap of proteins identified or quantified in three independent iTRAQ experiments and classification of the total proteins by GO categories. The *number* in *parentheses* indicates the number of identified proteins for each experiment. **A.** Overlap of proteins identified in three iTRAQ experiments. **B.** Overlap of proteins quantified in three iTRAQ experiments. Proteins were classified by GO biological process **C.**, molecular function **D.** and subcellular component **E.**

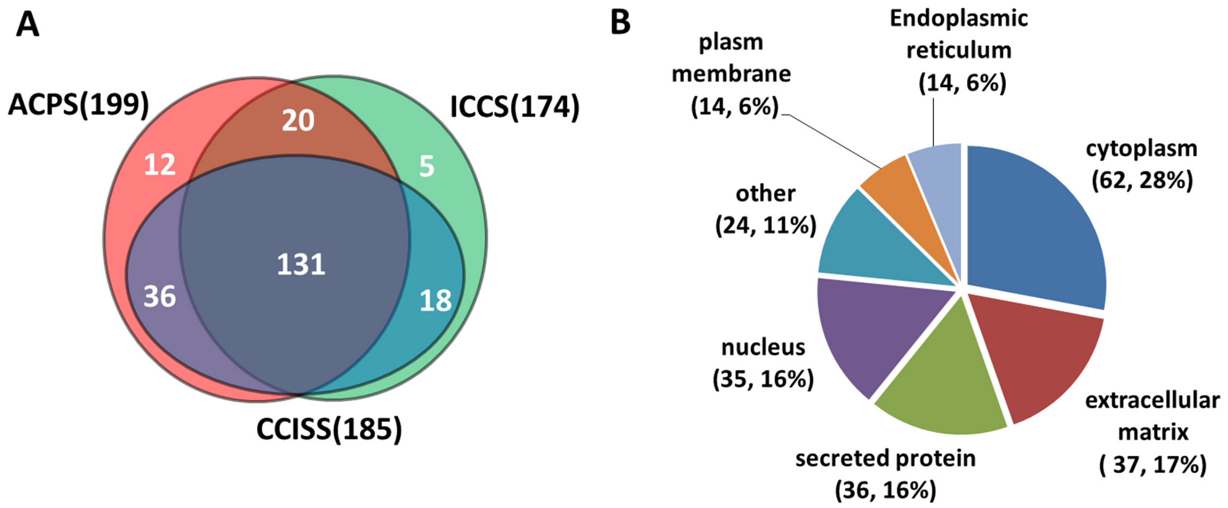

Supplementary Figure S3: Overlap of differentially expressed proteins in stroma at ACP, CIS and ICC stages of colorectal carcinogenesis process and their subcellular distribution. A. In total, 222 differentially expressed proteins were identified in stroma at ACP, CIS, or ICC vs NCM, among which 131 proteins (59%) differentially expressed proteins were common. Moreover, all of the differentially expressed proteins in ICC stages were included in ACP or CIS. **B.** Subcellular location was determined according to Uniprot database.

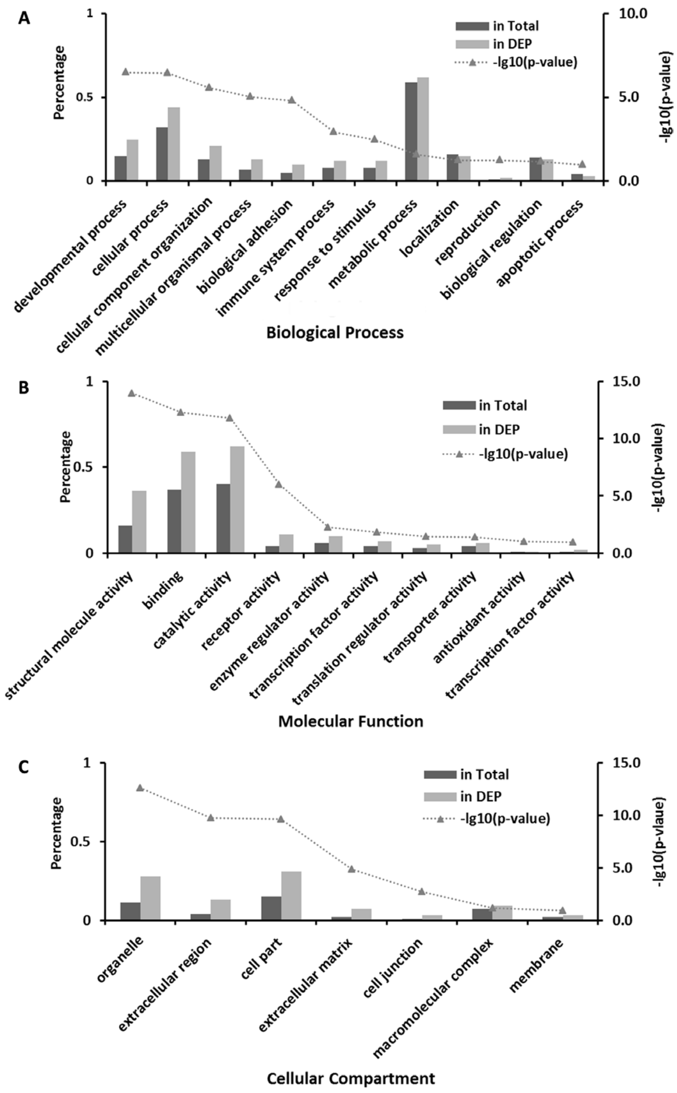

Supplementary Figure S4: GO enrichment analysis of differentially expressed proteins. A. Biological process, **B.** molecular function, **C.** subcellular compartment. Left axis: the percentage of the proteins in total dataset or in DEPs dataset belonging to the corresponding GO term category. Right axis: - log 10 of p-value (hypergeometric test, calculated by PANTHER)

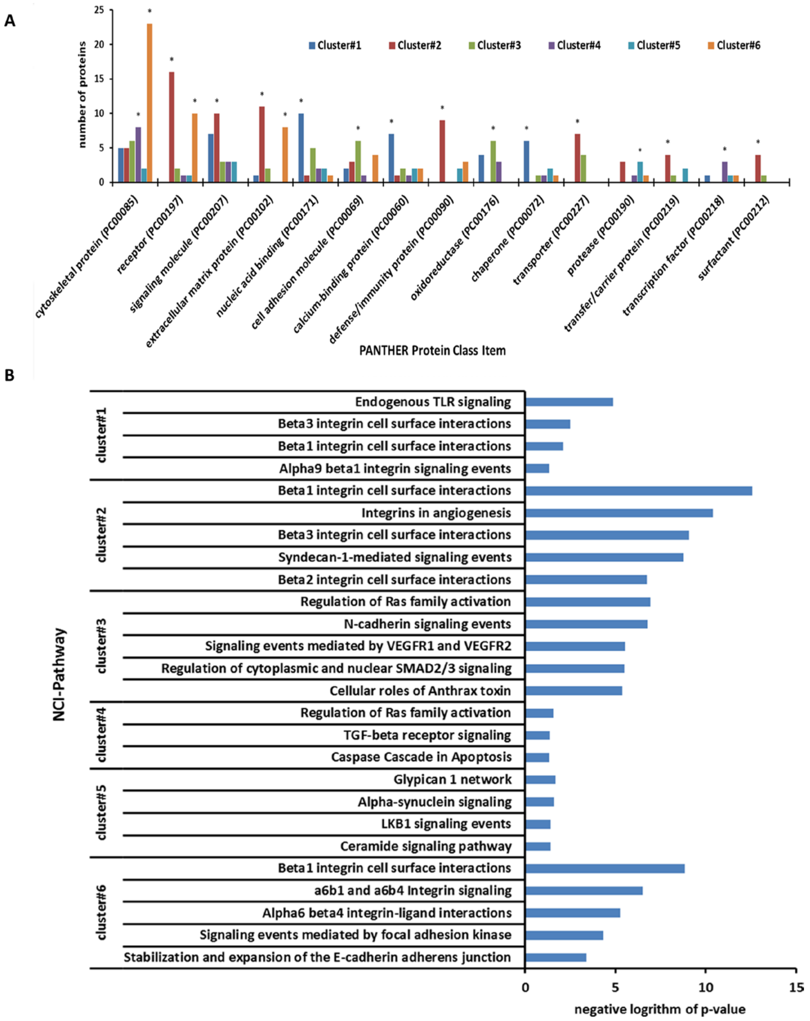

Supplementary Figure S5: PANTHER protein class enrichment analysis and NCI Pathway analysis of the differentially expressed proteins in each cluster. A. PANTHER protein class enrichment analysis for the proteins in each of the six K-mean clusters. * indicates that p-value <0.05 (hypergeometric test, total identified proteins as background). **B.** NCI-Pathway analysis for proteins in each clusters using NCI-nature Pathway interaction database.

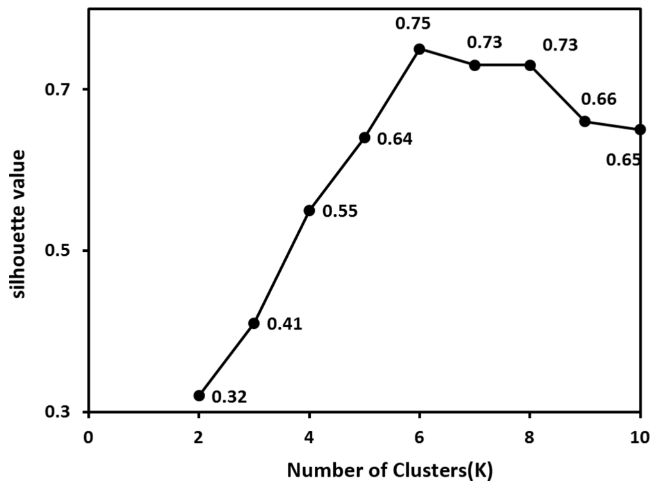

Supplementary Figure S6: Silhouette plot for Determination of the optimal number of clusters. Silhouette value was maximum at k=6.

Supplementary Table S1: Clinical features of tissue samples

Clinical features	iTRAQ	IHC
Gender (Male/Female)	14/8	79/64
Mean age	57±14	62±11
Age Range	24-80	24-86
Tissue state		
Adenomas	8(36%)	50(35%)
Carcinoma in situ(High-grade intraepithelial neoplasia)	5(23%)	30(21%)
Adenocarcinoma	9(41%)	63(44%)
Location		
Colon	19(86%)	128(91%)
Sigmoid	3(14%)	15(10%)

Supplementary Table S2: List of Differentially expressed proteins and K-mean clusters

See supplementary File 1
