# Supplementary material for: Proteomic analysis of stromal proteins in different stages of colorectal cancer establishes Tenascin-C as a stromal biomarker for colorectal cancer metastasis
Source: Oncotarget. 2016 May 14;7(24):37226–37. doi: 10.18632/oncotarget.9362 (PMC5095071; doi:10.18632/oncotarget.9362)
Supplement: Supplementary file 2 [file oncotarget-07-37226-s002.docx]

**Supplementary Table S3. List of Differentially expressed proteins and K-mean clusters**

| Cluster No. | No | Gene Symbol | ACP:NNCM | CIS:NNCM | ICC:NNCM | Subcellular* |
| --- | --- | --- | --- | --- | --- | --- |
| 1 | 1 | S100A9 | 10.26±1.63 | 7.13±2.21 | 18.71±2.84 | secreted |
| 1 | 2 | MYH9 | 3.94±0.8 | 11.12±1.62 | 13.51±2.44 | cytoplasm |
| 1 | 3 | TNC | 2.14±0.61 | 23.99±3.7 | 12.71±2.79 | ECM |
| 1 | 4 | HSPA5 | 8.42±1.83 | 6.56±1.09 | 10.81±1.95 | ER |
| 1 | 7 | EPX | 16.67±5.63 | 16.07±4.28 | 8.78±4.08 | cytoplasm |
| 1 | 8 | S100A8 | 9.35±4.71 | 6.14±3.02 | 8.63±4.33 | secreted |
| 1 | 9 | RRBP1 | 4.97±0.74 | 5.8±0.76 | 8.07±1.55 | ER |
| 1 | 10 | CKAP4 | 2.96±0.78 | 4.02±1.71 | 7.79±1.44 | ER |
| 1 | 12 | P4HB | 8.1±2.24 | 7.88±1.18 | 7.28±3.07 | cell membrane |
| 1 | 14 | PDIA3 | 4.56±1.3 | 2.21±0.62 | 6.03±1.66 | ER |
| 1 | 15 | LRRC59 | 4.74±3.79 | 4.11±2.16 | 5.68±3.69 | ER |
| 1 | 18 | PDIA4 | 4.33±1.65 | 1.57±0.94 | 5.34±1.96 | ER |
| 1 | 20 | RPN1 | 3.48±1.66 | 3.91±1.48 | 4.61±1.74 | ER |
| 1 | 21 | CANX | 4.46±2.41 | 3.65±1.81 | 4.56±1.84 | ER |
| 1 | 22 | PGD | 5.22±2.36 | 4.73±3.22 | 4.53±2.82 | cytoplasm |
| 1 | 23 | POSTN | 0.67±0.14 | 6.95±2.13 | 4.41±1.64 | ECM |
| 1 | 24 | HSPA8 | 3.23±0.59 | 2.44±0.9 | 4.42±1.28 | nucleus |
| 1 | 26 | TPM4 | 1±0.45 | 4.07±0.69 | 4.31±0.97 | cytoplasm |
| 1 | 27 | PKM | 1.68±0.2 | 2.4±0.27 | 4.18±0.64 | ECM |
| 1 | 30 | EEF1A1 | 6.59±1.31 | 5.35±1.45 | 4.03±0.65 | nucleus |
| 1 | 31 | TXNDC5 | 6.78±1.75 | 4.32±0.8 | 3.93±1.04 | ER |
| 1 | 32 | EEF2 | 5.86±1.23 | 6.05±0.93 | 3.86±1.02 | nucleus |
| 1 | 33 | MVP | 5.34±1.76 | 4.39±1.26 | 3.87±1.43 | nucleus |
| 1 | 35 | EEF1D | 2.75±0.97 | 4.42±1.37 | 3.81±1.94 | nucleus |
| 1 | 36 | HSP90B1 | 6.1±2.01 | 5.37±1.65 | 3.78±1.44 | ECM |
| 1 | 37 | HDLBP | 1.95±0.55 | 2.49±0.64 | 3.78±1.11 | nucleus |
| 1 | 38 | CALU | 1.74±0.88 | 3.18±1.87 | 3.48±1.31 | secreted |
| 1 | 39 | PLEC | 0.97±0.15 | 2.86±0.37 | 3.48±0.45 | cytoplasm |
| 1 | 44 | CALR | 5.67±2.18 | 2.14±0.78 | 3.05±1.38 | ECM |
| 1 | 46 | HYOU1 | 4.47±1.45 | 2.52±1.15 | 3±1.1 | ER |
| 1 | 47 | PABPC1 | 2.78±1.06 | 3.03±1.08 | 2.96±1.21 | nucleus |
| 1 | 49 | SND1 | 2.11±0.43 | 2.84±0.72 | 2.89±0.86 | nucleus |
| 1 | 50 | RCN1 | 3.44±1.46 | 2.94±1.18 | 2.84±0.95 | ER |
| 1 | 53 | RPL5 | 2.79±1.21 | 1.95±0.57 | 2.52±0.94 | nucleus |
| 1 | 54 | HSPD1 | 5.08±0.84 | 2.17±0.31 | 2.49±0.45 | cytoplasm |
| 1 | 56 | RPN2 | 3.28±1.36 | 2.95±1.17 | 2.34±0.87 | ER |
| 1 | 58 | LDHA | 3.97±2.98 | 6.08±4.36 | 2.27±0.81 | cytoplasm |
| 1 | 59 | EEF1B2 | 2.08±1.19 | 2.33±0.91 | 2.26±0.81 | cytoplasm |
| 1 | 61 | EMILIN1 | 0.74±0.21 | 2.78±0.9 | 2.16±0.79 | ECM |
| 1 | 64 | S100A11 | 4.88±2.01 | 2.41±0.98 | 2.05±0.61 | nucleus |
| 1 | 65 | FLNB | 2.5±0.53 | 2.45±0.38 | 1.95±0.41 | cytoplasm |
| 1 | 67 | COPA | 2.5±1.59 | 3.39±1.59 | 1.88±0.63 | cell membrane |
| 1 | 68 | FTL | 4.76±2.05 | 3.63±2.42 | 1.87±0.91 | ER |
| 1 | 72 | EEF1G | 3.8±1.33 | 4.43±1.91 | 1.72±0.3 | cytoplasm |
| 1 | 73 | TGFBI | 1.03±0.23 | 2.54±0.46 | 1.65±0.31 | ECM |
| 1 | 75 | RANGAP1 | 1.58±1.21 | 1.35±1.68 | 1.57±1.57 | cell membrane |
| 1 | 77 | CLTC | 2.36±0.34 | 2.14±0.34 | 1.4±0.6 | cell membrane |
| 1 | 80 | DHX9 | 2.89±0.47 | 2.49±0.45 | 1.32±0.23 | nucleus |
| 1 | 93 | HSP90AB1 | 2.13±0.82 | 3.01±0.97 | 1.1±0.71 | cytoplasm |
| 2 | 5 | FGB | 0.21±0.12 | 0.73±0.24 | 10.57±2.05 | secreted |
| 2 | 6 | FGG | 0.21±0.06 | 0.59±0.18 | 9.32±1.11 | secreted |
| 2 | 11 | FGA | 0.2±0.05 | 0.44±0.08 | 7.79±1.17 | secreted |
| 2 | 13 | CFB | 0.27±0.12 | 0.49±0.24 | 6.34±2.05 | secreted |
| 2 | 16 | MYH10 | 0.2±0.13 | 1.99±0.37 | 5.64±1.54 | cytoplasm |
| 2 | 17 | COL12A1 | 0.33±0.06 | 3.5±1.05 | 5.53±1.38 | ECM |
| 2 | 19 | IGLC2 | 0.82±0.14 | 0.81±0.14 | 4.88±0.75 | ER |
| 2 | 25 | BGN | 0.48±0.13 | 1.73±0.47 | 4.35±0.76 | ECM |
| 2 | 28 | CFH | 0.55±0.1 | 0.63±0.26 | 4.18±1.59 | secreted |
| 2 | 29 | C4A | 0.5±0.15 | 0.44±0.1 | 4.07±0.87 | secreted |
| 2 | 34 | HRG | 0.46±0.2 | 0.44±0.21 | 3.86±1.25 | secreted |
| 2 | 40 | FN1 | 0.12±0.04 | 2.33±0.34 | 3.37±0.65 | ECM |
| 2 | 41 | C3 | 0.16±0.04 | 0.38±0.1 | 3.32±0.49 | secreted |
| 2 | 42 | COL1A1 | 0.18±0.05 | 0.45±0.16 | 3.19±0.67 | ECM |
| 2 | 43 | KNG1 | 0.5±0.17 | 0.43±0.16 | 3.11±0.65 | secreted |
| 2 | 48 | COL1A2 | 0.18±0.04 | 0.56±0.11 | 2.93±0.55 | ECM |
| 2 | 51 | F13A1 | 0.26±0.08 | 0.56±0.14 | 2.83±0.33 | secreted |
| 2 | 52 | TF | 0.23±0.06 | 0.14±0.05 | 2.84±0.18 | secreted |
| 2 | 57 | HPX | 0.39±0.07 | 0.31±0.11 | 2.32±0.37 | secreted |
| 2 | 60 | SERPINA3 | 0.13±0.02 | 0.24±0.07 | 2.21±0.63 | secreted |
| 2 | 62 | ANXA1 | 0.31±0.06 | 0.6±0.1 | 2.14±0.37 | nucleus |
| 2 | 71 | APOA1 | 0.25±0.06 | 0.5±0.12 | 1.83±0.37 | secreted |
| 2 | 74 | LUM | 0.12±0.05 | 0.29±0.08 | 1.58±0.3 | ECM |
| 2 | 78 | VTN | 0.11±0.03 | 0.58±0.11 | 1.34±0.25 | ECM |
| 2 | 82 | DPYSL3 | 0.22±0.12 | 1.17±0.19 | 1.3±0.32 | cytoplasm |
| 2 | 83 | COL14A1 | 0.11±0.02 | 0.2±0.03 | 1.29±0.1 | ECM |
| 2 | 84 | ORM1 | 0.21±0.1 | 0.15±0.05 | 1.3±0.27 | secreted |
| 2 | 85 | SERPINA1 | 0.08±0.02 | 0.11±0.04 | 1.29±0.05 | ECM |
| 2 | 86 | FBLN1 | 0.24±0.06 | 1.2±0.22 | 1.28±0.43 | ECM |
| 2 | 87 | MSN | 0.25±0.14 | 0.73±0.13 | 1.27±0.42 | cell membrane |
| 2 | 88 | COL6A2 | 0.17±0.03 | 0.61±0.14 | 1.27±0.29 | ECM |
| 2 | 89 | VIM | 0.4±0.38 | 0.68±0.27 | 1.24±0.25 | cytoplasm |
| 2 | 90 | ITIH2 | 0.35±0.16 | 0.44±0.07 | 1.23±0.4 | secreted |
| 2 | 91 | AHSG | 0.26±0.1 | 0.28±0.1 | 1.23±0.28 | secreted |
| 2 | 94 | LMNA | 0.19±0.04 | 0.18±0.06 | 1.09±0.07 | nucleus |
| 2 | 96 | OGN | 0.08±0.03 | 0.07±0.03 | 1.05±0.15 | ECM |
| 2 | 99 | COL6A1 | 0.12±0.02 | 0.58±0.06 | 1.03±0.17 | ECM |
| 2 | 100 | DPYSL2 | 0.37±0.05 | 0.4±0.12 | 1.02±0.13 | cytoplasm |
| 2 | 101 | GSN | 0.27±0.04 | 0.2±0.09 | 0.99±0.04 | secreted |
| 2 | 102 | DCN | 0.12±0.02 | 0.18±0.05 | 0.98±0.17 | ECM |
| 2 | 105 | CLU | 0.09±0.05 | 0.5±0.26 | 0.94±0.18 | ECM |
| 2 | 106 | TNXB | 0.31±0.05 | 0.45±0.13 | 0.93±0.12 | ECM |
| 2 | 108 | AKAP12 | 0.18±0.1 | 0.5±0.08 | 0.92±0.31 | cytoplasm |
| 2 | 109 | MAP4 | 0.26±0.09 | 0.7±0.29 | 0.91±0.21 | cytoplasm |
| 2 | 110 | PRELP | 0.12±0.05 | 0.17±0.06 | 0.9±0.11 | ECM |
| 2 | 115 | COL6A3 | 0.07±0.02 | 0.27±0.05 | 0.8±0.07 | ECM |
| 2 | 123 | ASPN | 0.12±0.06 | 0.2±0.02 | 0.67±0.09 | ECM |
| 2 | 127 | AHNAK | 0.1±0.02 | 0.23±0.04 | 0.61±0.05 | nucleus |
| 3 | 76 | AGR2 | 10.62±3.23 | 1.43±0.62 | 1.45±0.54 | secreted |
| 3 | 81 | KRT20 | 6.73±0.77 | 2.97±0.64 | 1.3±0.3 | cytoplasm |
| 3 | 97 | CDH17 | 3.33±1.08 | 1.23±0.26 | 1.04±0.37 | ER |
| 3 | 103 | SERPINB1 | 2.51±0.53 | 1.64±0.44 | 0.96±0.29 | cytoplasm |
| 3 | 111 | GANAB | 2.75±0.59 | 1.71±0.35 | 0.87±0.22 | ER |
| 3 | 112 | LRPPRC | 2.84±1.16 | 3.59±0.71 | 0.87±0.43 | nucleus |
| 3 | 117 | CTNNA1 | 2.35±0.35 | 1.19±0.31 | 0.78±0.38 | cell membrane |
| 3 | 128 | IDH2 | 5.7±2.61 | 3.07±2.12 | 0.59±0.36 | ER |
| 3 | 133 | PSME1 | 3.39±0.88 | 1.06±0.32 | 0.54±0.3 | cytoplasm |
| 3 | 134 | ENO1 | 2.3±0.31 | 1.35±0.2 | 0.53±0.13 | cytoplasm |
| 3 | 136 | FH | 1.2±0.3 | 0.56±0.16 | 0.53±0.08 | cytoplasm |
| 3 | 137 | TALDO1 | 1.13±0.1 | 0.72±0.2 | 0.52±0.13 | cytoplasm |
| 3 | 138 | TST | 2.18±0.18 | 0.46±0.12 | 0.51±0.17 | ER |
| 3 | 143 | MDH2 | 1.71±0.4 | 0.52±0.11 | 0.47±0.14 | ER |
| 3 | 145 | MUC2 | 1.46±0.23 | 0.37±0.07 | 0.47±0.15 | secreted |
| 3 | 148 | ATP5B | 1.44±0.26 | 0.43±0.05 | 0.46±0.08 | ER |
| 3 | 156 | ACAA2 | 2.45±0.92 | 0.55±0.24 | 0.41±0.18 | ER |
| 3 | 158 | FCGBP | 2.23±0.19 | 0.71±0.08 | 0.39±0.1 | secreted |
| 3 | 163 | ACTN4 | 1±0.33 | 1.87±0.42 | 0.36±0.11 | nucleus |
| 3 | 164 | LGALS3 | 1.27±0.32 | 0.66±0.16 | 0.35±0.19 | ECM |
| 3 | 166 | KRT18 | 4.9±0.8 | 2.53±0.25 | 0.34±0.08 | nucleus |
| 3 | 173 | KRT8 | 4.82±0.53 | 2.42±0.29 | 0.29±0.12 | nucleus |
| 3 | 175 | GAPDH | 1.02±0.12 | 0.36±0.16 | 0.29±0.03 | nucleus |
| 3 | 176 | VDAC1 | 2.14±1.02 | 0.86±0.53 | 0.28±0.33 | ER |
| 3 | 179 | CLCA1 | 7.37±1.92 | 1.26±0.72 | 0.26±0.1 | secreted |
| 3 | 180 | PYGB | 1.27±0.42 | 0.71±0.31 | 0.26±0.26 | cytoplasm |
| 3 | 183 | PPIA | 1.94±0.38 | 0.51±0.1 | 0.25±0.07 | secreted |
| 3 | 184 | HADH | 1.67±0.44 | 0.61±0.2 | 0.25±0.1 | cytoplasm |
| 3 | 192 | LGALS4 | 3.75±0.76 | 0.99±0.25 | 0.2±0.08 | ER |
| 3 | 193 | KRT19 | 2.76±0.56 | 1.21±0.34 | 0.2±0.08 | ER |
| 3 | 194 | PFN1 | 0.76±0.08 | 0.73±0.18 | 0.19±0.05 | cytoplasm |
| 3 | 200 | COX5B | 0.91±0.18 | 0.56±0.22 | 0.18±0.08 | ER |
| 3 | 202 | YWHAZ | 1.02±0.18 | 0.97±0.12 | 0.17±0.05 | cytoplasm |
| 3 | 204 | S100A6 | 1.84±0.29 | 0.59±0.1 | 0.17±0.08 | cell membrane |
| 3 | 206 | PSAP | 0.43±0.09 | 0.83±0.16 | 0.15±0.05 | secreted |
| 3 | 207 | TXN | 1.58±0.45 | 0.63±0.13 | 0.15±0.11 | secreted |
| 3 | 217 | CALM1 | 1.13±0.2 | 0.55±0.15 | 0.12±0.06 | cytoplasm |
| 3 | 220 | HIST1H1D | 1.51±0.24 | 0.52±0.18 | 0.08±0.05 | nucleus |
| 3 | 221 | HIST1H1C | 1.93±0.28 | 0.62±0.05 | 0.06±0.05 | nucleus |
| 3 | 222 | CKB | 0.42±0.04 | 0.16±0.03 | 0.05±0.02 | cytoplasm |
| 3 | 223 | HIST1H1E | 1.15±0.16 | 0.49±0.07 | 0.02±0.01 | nucleus |
| 4 | 116 | SEPT7 | 0.44±0.08 | 0.55±0.1 | 0.79±0.4 | cytoplasm |
| 4 | 121 | SEPT2 | 0.35±0.11 | 0.43±0.12 | 0.67±0.13 | cytoplasm |
| 4 | 126 | LMNB2 | 0.29±0.1 | 0.26±0.04 | 0.62±0.16 | nucleus |
| 4 | 140 | PRDX6 | 0.73±0.2 | 0.45±0.09 | 0.51±0.21 | cytoplasm |
| 4 | 141 | VAT1 | 0.42±0.17 | 0.25±0.13 | 0.49±0.17 | cell membrane |
| 4 | 146 | RSU1 | 0.22±0.11 | 0.37±0.09 | 0.47±0.11 | ER |
| 4 | 149 | MYO1C | 0.44±0.12 | 0.42±0.14 | 0.44±0.16 | nucleus |
| 4 | 151 | ANXA5 | 0.16±0.04 | 0.29±0.11 | 0.43±0.14 | cytoplasm |
| 4 | 153 | LGALS1 | 0.25±0.08 | 0.3±0.07 | 0.43±0.17 | ECM |
| 4 | 155 | SPTAN1 | 0.45±0.07 | 0.34±0.08 | 0.41±0.05 | cytoplasm |
| 4 | 157 | SPTBN1 | 0.45±0.12 | 0.44±0.12 | 0.4±0.05 | cytoplasm |
| 4 | 168 | DCD | 0.27±0.08 | 0.1±0.08 | 0.33±0.09 | secreted |
| 4 | 171 | HSPA1A | 0.38±0.09 | 0.29±0.06 | 0.32±0.09 | ER |
| 4 | 178 | H1F0 | 0.28±0.07 | 0.12±0.06 | 0.27±0.13 | nucleus |
| 4 | 186 | ADH5 | 0.33±0.08 | 0.36±0.15 | 0.24±0.04 | cytoplasm |
| 4 | 187 | SDPR | 0.14±0.09 | 0.1±0.04 | 0.22±0.08 | cytoplasm |
| 4 | 189 | CHGA | 0.09±0.14 | 0.07±0.05 | 0.22±0.22 | secreted |
| 4 | 190 | PTRF | 0.05±0.01 | 0.08±0.03 | 0.22±0.04 | ER |
| 4 | 216 | TPSAB1 | 0.16±0.07 | 0.09±0.03 | 0.12±0.04 | secreted |
| 4 | 218 | FHL1 | 0.09±0.03 | 0.12±0.02 | 0.12±0.06 | nucleus |
| 4 | 219 | PRPH | 0.04±0.02 | 0.09±0.03 | 0.09±0.02 | ER |
| 5 | 45 | HP | 0.16±0.03 | 0.04±0 | 3.06±0.4 | secreted |
| 5 | 55 | HSPE1 | 2.79±0.59 | 0.53±0.18 | 2.48±0.57 | ER |
| 5 | 63 | LMNB1 | 2.56±0.45 | 0.73±0.26 | 2.07±0.65 | nucleus |
| 5 | 66 | HBA1 | 1.45±0.08 | 0.07±0.01 | 1.91±0.07 | ER |
| 5 | 69 | PGK1 | 2.23±0.31 | 0.54±0.11 | 1.86±0.7 | cytoplasm |
| 5 | 70 | PARK7 | 1.53±0.54 | 0.4±0.09 | 1.85±0.49 | nucleus |
| 5 | 79 | SERPINC1 | 0.59±0.11 | 0.18±0.08 | 1.33±0.16 | secreted |
| 5 | 92 | ANXA2 | 0.32±0.06 | 0.17±0.05 | 1.19±0.27 | ECM |
| 5 | 95 | HSPA9 | 2.23±0.57 | 0.68±0.25 | 1.06±0.25 | nucleus |
| 5 | 107 | KRT1 | 0.61±0.14 | 0.34±0.13 | 0.93±0.16 | ECM |
| 5 | 113 | A2M | 0.22±0.02 | 0.13±0.03 | 0.86±0.11 | secreted |
| 5 | 118 | RPS27A | 0.64±0.09 | 0.15±0.05 | 0.73±0.2 | nucleus |
| 5 | 122 | EPB41L2 | 0.51±0.12 | 0.28±0.09 | 0.67±0.25 | cytoplasm |
| 5 | 129 | CTSD | 0.97±0.39 | 0.29±0.08 | 0.57±0.16 | secreted |
| 5 | 130 | CA1 | 0.58±0.14 | 0.2±0.15 | 0.56±0.26 | cytoplasm |
| 5 | 139 | CA2 | 0.71±0.19 | 0.15±0.01 | 0.51±0.1 | cytoplasm |
| 5 | 144 | S100A10 | 0.64±0.14 | 0.15±0.1 | 0.47±0.12 | ER |
| 5 | 165 | HBB | 0.86±0.14 | 0.06±0.02 | 0.35±0.09 | ER |
| 6 | 104 | HSPG2 | 0.33±0.05 | 1.46±0.18 | 0.96±0.17 | ECM |
| 6 | 114 | PALLD | 0.18±0.04 | 1.07±0.11 | 0.84±0.27 | cytoplasm |
| 6 | 119 | NES | 0.29±0.07 | 0.65±0.2 | 0.71±0.1 | cytoplasm |
| 6 | 120 | NID2 | 0.25±0.08 | 0.55±0.26 | 0.69±0.22 | ECM |
| 6 | 124 | MYH11 | 0.62±0.06 | 0.75±0.04 | 0.67±0.05 | ER |
| 6 | 125 | TES | 0.37±0.17 | 0.68±0.25 | 0.64±0.14 | cytoplasm |
| 6 | 131 | CPA3 | 0.21±0.07 | 0.43±0.08 | 0.55±0.11 | cytoplasm |
| 6 | 132 | LAMB1 | 0.35±0.14 | 1.24±0.23 | 0.55±0.24 | ECM |
| 6 | 135 | TLN1 | 0.14±0.03 | 0.8±0.11 | 0.53±0.06 | cell membrane |
| 6 | 142 | KCTD12 | 0.28±0.12 | 0.57±0.22 | 0.47±0.37 | ER |
| 6 | 147 | NID1 | 0.29±0.05 | 0.47±0.09 | 0.47±0.09 | ECM |
| 6 | 150 | LAMC1 | 0.23±0.06 | 0.74±0.16 | 0.44±0.13 | ECM |
| 6 | 152 | ACTN1 | 0.17±0.05 | 1.13±0.19 | 0.43±0.14 | cytoplasm |
| 6 | 154 | ANXA6 | 0.15±0.05 | 0.48±0.06 | 0.42±0.12 | cytoplasm |
| 6 | 159 | FLNA | 0.1±0.31 | 0.59±0.16 | 0.39±0.24 | cytoplasm |
| 6 | 160 | TNS1 | 0.2±0.03 | 0.63±0.24 | 0.37±0.14 | cytoplasm |
| 6 | 161 | FLNC | 0.13±0.03 | 0.73±0.11 | 0.36±0.08 | cell membrane |
| 6 | 162 | CALD1 | 0.03±0.01 | 0.29±0.09 | 0.36±0.02 | cytoplasm |
| 6 | 167 | WDR1 | 0.4±0.15 | 0.8±0.22 | 0.34±0.08 | cytoplasm |
| 6 | 169 | ITGB1 | 0.46±0.08 | 0.97±0.23 | 0.33±0.14 | cytoplasm |
| 6 | 170 | FERMT2 | 0.13±0.08 | 0.66±0.2 | 0.32±0.11 | cell membrane |
| 6 | 172 | HSPB1 | 0.13±0.03 | 0.58±0.09 | 0.3±0.06 | nucleus |
| 6 | 174 | LAMB2 | 0.22±0.04 | 0.57±0.15 | 0.29±0.08 | ECM |
| 6 | 177 | LAMA4 | 0.22±0.09 | 0.61±0.2 | 0.27±0.09 | ECM |
| 6 | 181 | PARVA | 0.17±0.07 | 0.52±0.16 | 0.26±0.1 | cell membrane |
| 6 | 182 | APCS | 0.04±0.01 | 0.14±0.02 | 0.25±0.11 | secreted |
| 6 | 185 | SMTN | 0.15±0.06 | 0.44±0.07 | 0.24±0.09 | cytoplasm |
| 6 | 188 | SYNPO2 | 0.11±0.05 | 0.26±0.05 | 0.22±0.05 | nucleus |
| 6 | 191 | SYNM | 0.08±0.03 | 0.39±0.11 | 0.21±0.09 | cytoplasm |
| 6 | 195 | VCL | 0.09±0.01 | 0.38±0.03 | 0.19±0.01 | cell membrane |
| 6 | 196 | SORBS1 | 0.1±0.01 | 0.26±0.03 | 0.18±0.06 | nucleus |
| 6 | 197 | TAGLN | 0.04±0 | 0.55±0.04 | 0.18±0.02 | cytoplasm |
| 6 | 198 | LPP | 0.11±0.04 | 0.49±0.1 | 0.18±0.04 | nucleus |
| 6 | 199 | MYLK | 0.08±0.02 | 0.39±0.04 | 0.18±0.05 | cytoplasm |
| 6 | 201 | DES | 0.08±0.29 | 0.33±0.24 | 0.17±0.28 | cytoplasm |
| 6 | 203 | AOC3 | 0.24±0.07 | 0.36±0.19 | 0.17±0.04 | cytoplasm |
| 6 | 205 | PDLIM7 | 0.14±0.03 | 0.7±0.14 | 0.16±0.21 | cytoplasm |
| 6 | 208 | EHD2 | 0.13±0.04 | 0.26±0.06 | 0.15±0.08 | cell membrane |
| 6 | 209 | CNN1 | 0.03±0.01 | 0.26±0.07 | 0.15±0.03 | ER |
| 6 | 210 | CSRP1 | 0.13±0.05 | 0.49±0.06 | 0.14±0.03 | nucleus |
| 6 | 211 | PGM5 | 0.16±0.03 | 0.36±0.07 | 0.13±0.08 | cytoplasm |
| 6 | 212 | TGM2 | 0.05±0.02 | 0.24±0.04 | 0.13±0.03 | ER |
| 6 | 213 | MYL6 | 0.15±0.01 | 0.38±0.05 | 0.13±0.02 | ER |
| 6 | 214 | TPM2 | 0.04±0.01 | 0.43±0.08 | 0.13±0.02 | cytoplasm |
| 6 | 215 | TPM1 | 0.04±0.03 | 0.43±0.1 | 0.12±0.08 | cytoplasm |

*ECM: extracellular matrix; ER: endoplasmic reticulum
